# IQGAP1 Functions as a Modulator of Dishevelled Nuclear Localization in Wnt Signaling

**DOI:** 10.1371/journal.pone.0060865

**Published:** 2013-04-05

**Authors:** Toshiyasu Goto, Atsushi Sato, Masahiro Shimizu, Shungo Adachi, Kiyotoshi Satoh, Shun-ichiro Iemura, Tohru Natsume, Hiroshi Shibuya

**Affiliations:** 1 Department of Molecular Cell Biology, Medical Research Institute, Tokyo Medical and Dental University, Bunkyo-ku, Tokyo, Japan; 2 Biomedicinal Information Research Center, National Institutes of Advanced Industrial Science and Technology, Kohtoh-ku, Tokyo, Japan; National Cancer Center, Japan

## Abstract

Dishevelled (DVL) is a central factor in the Wnt signaling pathway, which is highly conserved among various organisms. DVL plays important roles in transcriptional activation in the nucleus, but the molecular mechanisms underlying their nuclear localization remain unclear. In the present study, we identified IQGAP1 as a regulator of DVL function. In *Xenopus* embryos, depletion of IQGAP1 reduced Wnt-induced nuclear accumulation of DVL, and expression of Wnt target genes during early embryogenesis. The domains in DVL and IQGAP1 that mediated their interaction are also required for their nuclear localization. Endogenous expression of Wnt target genes was reduced by depletion of IQGAP1 during early embryogenesis, but notably not by depletion of other IQGAP family genes. Moreover, expression of Wnt target genes caused by depletion of endogenous IQGAP1 could be rescued by expression of wild-type IQGAP1, but not IQGAP1 deleting DVL binding region. These results provide the first evidence that IQGAP1 functions as a modulator in the canonical Wnt signaling pathway.

## Introduction

Wnt signaling plays important roles in multiple developmental events during embryogenesis [1], [2]. Canonical Wnt signaling is initiated by binding of the Wnt ligand to the cell-surface Frizzled and transmembrane LRP complex. This leads to the membrane recruitment and activation of Dishevelled (DVL), which inactivates the APC/Axin/GSK-3 complex in the cytoplasm, responsible for the degradation of ß-catenin [3], [4]. As a result, ß-catenin accumulates in the cytoplasm, translocates to the nucleus and associates with Tcf transcription factors, which activate the Wnt target genes [5], [6]. In *Xenopus*, Wnt signaling accompanied by ß-catenin nuclear localization at the dorsal side is an important for axis formation during early embryogenesis [7]. Ventral over-expression of *Xwnt-8*, *ß-catenin* and *DVL2* induces a secondary axis and promotes expression of Wnt target genes, such as *Siamois*, *Xnr3* and *Xtwn* [8]–[14].

There are three DVL isoforms, DVL1, DVL2 and DVL3 [15], [16], which are well-conserved among various organisms. Each isoform plays a similar role in the canonical Wnt pathway, but have different sensitivities to Wnt stimulation [17]. DVL contains three conserved regions known as the DIX, PDZ and DEP domains [18], [19]. Both the DIX and PDZ domains are necessary for canonical Wnt inactivation of ß-catenin degradation [20], [21]. In contrast, the DEP domain does not affect canonical signaling, but is involved in the planar cell polarity (PCP) pathway [21]–[23]. DVL plays an additional role in the Wnt signaling pathway, by localizing to the nucleus and binding a complex containing ß-catenin and Tcf, which in turn activates Wnt target genes in the nucleus [24]. The subcellular localization of DVL, either on the cell membrane or in the nucleus, is important for understanding its function in Wnt signaling.

IQGAP1 contains multiple protein-interacting domains: the CH (calponin homology) domain binds to F-actin, the WW domain binds to ERK2, the IQ repeat motifs bind to calmodulin and myosin light chain, and the Ras GAP-like domain binds to Cdc42 and Rac1 [25]–[32]. IQGAP1 is also known to bind to E-cadherin and ß-catenin, and is involved in cytoskeltal reorganization and cell adhesion [33], [34]. On the other hand, IQGAP1 stimulates ß-catenin-mediated transcriptional activation^34^. The subcellular localization of IQGAP1 varies in several cultured cells, and analysis of its domains indicates that IQGAP1 may be localized in the cytoplasm, cell membrane and nucleus [35]. These subcellular localizations are presumably linked to its cellular functions. There are also three isoforms of IQGAP: IQGAP1, IQGAP2 and IQGAP3. Their subcellular localizations suggest both similarities and differences in function [36]. Each isoform has a different role, and in some cases IQGAP1 has an opposite function to IQGAP2 [36], [37]. The *Xenopus xIQGAP1* and *xIQGAP2* genes have been isolated [38] and shown to be involved in cadherin-mediated cell adhesion [38], [39]. We also isolated *xIQGAP3* and have generated antisense morpholino oligonucleotides based on these sequences.

In the present study, we identified IQGAP1 as a novel DVL-binding protein. Binding between IQGAP1 and DVL2 mutually contributed to their nuclear localization. The depletion of endogenous IQGAP1 in *Xenopus* embryos suppressed secondary axis induction and expression of Wnt target genes. These results reveal a novel role for IQGAP1 in modulating the subcellular localization and transcriptional activation of components of the Wnt signaling pathway.

## Materials and Methods

### Ethics statement

All animal experiments were performed under the ethical guidelines of Tokyo Medical and Dental University, and animal protocols were reviewed and approved by the animal welfare committee of the Tokyo Medical and Dental University.

### Plasmid construction

The human and *Xenopus DVL*, *IQGAP* isoforms were amplified by RT-PCR from cDNA templates prepared from HEK 293 cells and *Xenopus* embryos, respectively, and were subcloned into the pRK5 and pCS2+ vectors. Each truncated mutant was constructed by PCR and contained the following amino acid sequences. hDVL1-1: 1–486 aa, hDVL1-2: 476–671 aa, hIQGAP1-1: 1–669 aa, hIQGAP1-2: 631–951 aa, hIQGAP1-3: 631–1657 aa, hIQGAP1-4: 901–1060 aa, hIQGAP1-5: 1067–1154 aa, hIQGAP1-6: 1032–1116 aa, xDVL2-*Δ*IBR: 1–509 aa, xDVL2-IBR: 510–736 aa, xIQGAP1-*Δ*DBR: 1–856 and 1061–1657 aa, xIQGAP1-DBR: 857–1060 aa. We made GFP constructs of xDVL2, xIQGAP1 and their truncated mutants by conjugating with the GFP sequence at the C-terminus.

### Primers

The sequences of the primer pairs were as follows. In Figure S1, xDVL1: Forward 5′-CCAGCATAGCGAAGGTAGTA-3′; Reverse 5′-TACACCTTGCTCCCGATCTT-3′. xDVL2: Forward 5′-ATCTGACTGGCTGTGAGAAC-3′; Reverse 5′-TCAGACTCACTACCAGATCC-3′. xDVL3: Forward 5′-AAGTCTGGAGGAAGTGGAAG-3′; Reverse 5′-CATGCGGAAGGATTGTCTAC-3′. xIQGAP1: Forward 5′-CAGTGAACAGGAAGCAGATC-3′; Reverse 5′-TCAATGCTGTGTGTGTCTGC-3′. xIQGAP2: Forward 5′-CAGAAGAAAAGGGCTCCAAG-3′; Reverse 5′-AACATCTTCATCACGGCGAC-3′. xIQGAP3: Forward 5′-ACAGCCAACTTAGCATCGAG-3′; Reverse 5′-TGCTGTGTAATTGAGGGACG-3′. Ornithine decarboxylase (ODC): Forward 5′-GTCAATGATGGAGTGTATGGATC-3′; Reverse 5′-TCCATTCCGCTCTCCTGAGCAC-3′. IIn Figure S5, Glyceraldehyde-3-phosphate dehydrogenase (GAPDH): Forward 5′-GCCATCACTGCCACCCAGAAGACTG-3′; Reverse 5′-CATGAGGTCCACCACCCTGTTGCTG-3′. Axin2: Forward 5′-AACGACAGCGAGTTATCCAGCGACG-3′; Reverse 5′-ATGACACTGCTGATGGTGGTGGTGC-3′. TGFß2: Forward 5′-TGGCTTCACCATAAAGACAGGAACC-3′; Reverse 5′-CAGAAGTTGGCATTGTACCCTTTGG-3′. Mouse IQGAP1: Forward 5′-AAGTTTGACGTGCCTGGTGA-3′; Reverse 5′-GGTATCTGTTCTTTGGGTCC-3′. xIQGAP1: Forward 5′-AGCTTGCAGATATGATGATG-3′; Reverse 5′-TTAGTCCACAGAGCTAATGATG-3′.

### Embryo handling and morpholino oligonucleotides

Capped mRNAs were synthesized from linearized vectors using the mMessage Machine kit (Ambion). The morpholino oligonucleotides (MO) (Gene Tools, LLC) used here were 5′-CCTCTTACCTCAGTTACAATTTATA-3′ (Control-MO), 5′-GTAGATGATTTTGGTCTCAGCCATG-3′ (*xDVL1*-MO), 5′-TCACTTTAGTCTCCGCCATTCTGCG-3′ (*xDVL2*-MO), 5′-GATGACCTTGGTCTCCCCCATAATT-3′ (*xDVL3*-MO), 5′-CATCGACTTCCTCCGAAACGGACAT-3′ (*xIQGAP1*-MO), 5′-GTCCTCATGGTTCATCCTGTTGCTG-3′ (*xIQGAP2*-MO), 5′-CCTCCGGCCTTACACTCCATTCCTG-3′ (*xIQGAP3*-MO). The specificity of each MO was confirmed by its ability to inhibit the translation of FLAG-tagged mRNAs containing the targeted site with or without 5-mismatched sequences. MO (10 ng) and FLAG-tagged mRNAs (100 pg) were co-injected with *ß-globin-FLAG* mRNA (100 pg) as loading control into the animal poles of 4-cell stage embryos, and the injected animal caps were dissected at stage 10. Lysates from the animal caps were subjected to Western blotting with anti-FLAG antibody (M2, Sigma) (Fig. S1C).

MOs and mRNAs were injected into four animal blastomeres at the 4-cell stage for dissection of animal caps or into two dorsal or ventral blastomeres at the 4-cell-stage for quantitative RT-PCR analysis and observation of embryo phenotypes. Animal cap explants of the injected (10 pg mRNA of each GFP fused construct) embryos were dissected at the early gastrula stage (st.10), and fixed for DAPI staining as previously reported [40]. We counted the number of cell that has fluorescence signals. When the fluorescence signal overlapped with DAPI staining was similar and brighter than un-overlapped fluorescence signal in counted cells, we defined such cells as nuclear localized cells. If nuclear fluorescence signals were not clear, we used ImageJ software (NIH) and measured the strength of brightness of fluorescence signals to define nuclear localized signals or not. The ratio of nuclear localized cells in total counted cells was computed for every explant and the average of ratio was taken with six explants in 3 independent experiments. Dorsal or ventral sectors of the injected embryos were dissected at st.10, and total RNA was extracted for RT-PCR analysis. The cytoplasmic and nuclear fractions were prepared as described with modifications [41].

### RT-PCR analysis

Total RNA was prepared using TRIzol (Invitrogen). cDNA synthesis was carried out using Moloney murine leukemia virus reverse transcriptase (Invitrogen). Quantitative PCR was performed with an Applied Biosystems 7300 Real-Time PCR Cycler (ABI) using THUNDERBIRD SYBR qPCR Mix (TOYOBO). The sequences of the primer pairs were as follows. *Ornithine decarboxylase (ODC)*: Forward 5′-AAAATGGATGACTGCGAGATGGG-3′; Reverse 5′-AATGAAGATGCTGACTGGCAAAAC-3′. *Siamois*: Forward 5′-CTGTCCTACAAGAGACTCTG-3′; Reverse 5′-TGTTGACTGCAGACTGTTGA-3′. *Xnr3*: Forward 5′-CTTCTGCACTAGATTCTG-3′; Reverse 5′-CAGCTTCTGGCCAAGACT-3′. *Xtwn*: Forward 5′-AACCCAAGAAGGCGACACTATC-3′; Reverse 5′-GTGCCGATGGTAGGAAATGATC-3′. *Xenopus* embryonic *ODC* was used for normalization of cDNA samples.

### Antibodies and cell lines

The following antibodies were used for immunoprecipitation and/or Western blotting analysis: Horseradish peroxidase conjugated anti-mouse IgG (GE); Horseradish peroxidase conjugated anti-rabbit IgG (GE); anti-FLAG (M2 and F7425, Sigma); anti-MYC (9B11, Cell Signaling); anti-DVL1 (3F12 and Q-25, Santa Cruz); anti-IQGAP1 (H-109, Santa Cruz); anti-beta-tubulin (sc-58884, Santa Cruz); anti-histone-H3 (sc-10809, Santa Cruz). We used following cell lines: HEK 293 cells [42], HEK 293T cells [43], NIH3T3 cells [43], L cells (CRL-2648, ATCC), L Wnt3A cells (CRL-2647, ATCC). Recombinant human Wnt3A (R&D Systems; 20 ng/ml) or four day Wnt-3A conditioned medium from L-Wnt-3A cells was used for Wnt stimulation of cultured cells. The growth medium for each cell type is described by American Type Culture Collection.

### Protein identification by LC-MS/MS analysis

FLAG-human DVL1 was expressed in HEK 293 cells, and DVL1 and associated proteins were recovered from cell extracts by immunoprecipitation with anti-FLAG antibody. The DVL1-associated complexes were digested with *Axhromobacter* protease I, and the resulting peptides were analyzed using a nanoscale LC-MS/MS system, as described previously [44].

## Results

### IQGAP associates with DVL

**Figure 1:**
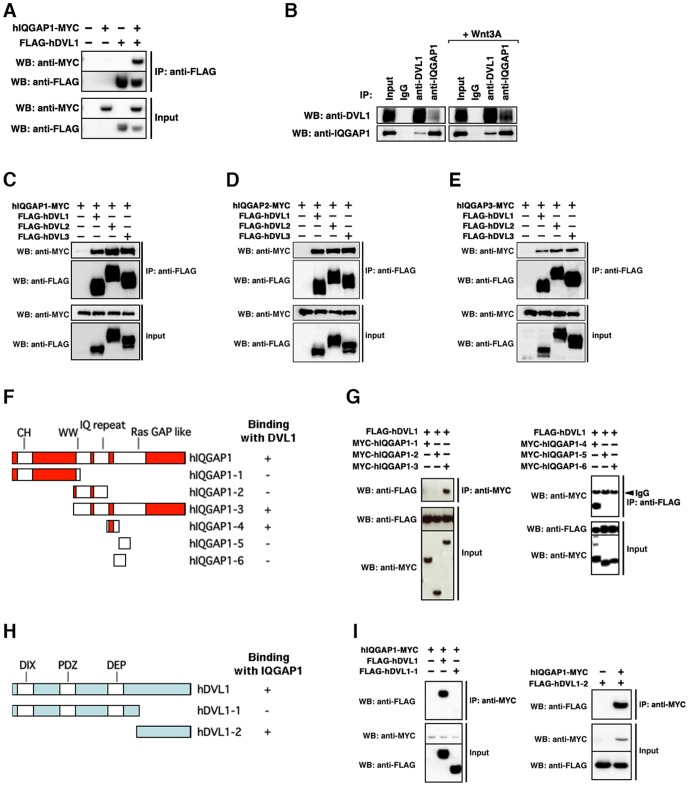
IQGAP associates with DVL. (**A**) Interaction between ectopically-expressed hIQGAP1 and hDVL1 in HEK 293 cells. Immunoprecipitates (IP) obtained using anti-FLAG antibody were subjected to Western blotting (WB) with the indicated antibodies. +, present; -, absent. (**B**) Interaction between endogenous hIQGAP1 and hDVL1 in HEK 293T cells. The cultured cells were stimulated with recombinant human Wnt3A for 6 hours (right panels). (**C–E**) Interaction between ectopically-expressed hIQGAP1, hIQGAP2, hIQGAP3 and hDVL isoforms in HEK 293T cells. (C) hIQGAP1. (D) hIQGAP2. (E) hIQGAP3. (**F**) A schematic of the domains of hIQGAP1 and truncated constructs. (**G**) Interactions between ectopically-expressed hDVL1 and truncated hIQGAP1 constructs. (**H**) Interactions among ectopically-expressed hIQGAP1 and truncated hDVL1 constructs. (**I**) A schematic of the domains of hDVL1 and truncated constructs.

To identify novel proteins that may bind to DVL, we performed a high-throughput analysis of proteins that co-immunoprecipitated with mouse DVL1 in HEK 293 cells using direct nanoflow liquid chromatography-coupled tandem mass spectrometry [44]. We identified several known DVL-binding proteins, such as CK1 [45], CK2 [46], Strabismus [47], Par1 [48], Axin [49] and PP2C [50]. In addition, we identified IQGAP1 as a candidate protein that may physically interact with DVL1. An interaction between ectopically expressed IQGAP1 and DVL1 was confirmed in HEK 293 cells (Fig. 1A). Immunoprecipitation analysis using each protein antibody also confirmed the existence of an endogenous IQGAP1 and DVL1 complex in HEK 293T cells, and their interaction was increased by Wnt stimulation (Fig. 1B). In vertebrates, three isoforms of IQGAP and DVL have been identified: IQGAP1, IQGAP2 and IQGAP3, and DVL1, DVL2 and DVL3. We confirmed that each IQGAP isoform also bound to each DVL isoform (Fig. 1C-1E). To determine the region in IQGAP1 responsible for binding to DVL1, several truncated mutants of IQGAP1 were examined in co-precipitation assays. We found that the region between the C-terminal IQ repeat domain and the N-terminal Ras GAP-like domain of IQGAP1 (termed DBR; Dishevelled Binding Region) was responsible for binding to DVL1 (Fig. 1F and 1G) Conversely, the C-terminus of DVL1 (termed IBR; IQGAP Binding Region) is necessary for binding to IQGAP1 (Fig. 1H and 1I). Both the IBR and DBR are well-conserved among the three DVL and IQGAP isoforms, respectively. The amino acid sequence similarities of IBR and DBR among the three DVL and IQGAP isoforms were as follows, the similarity of IBR between DVL1 and DVL2 is 47.1%; the similarity of IBR between DVL1 and DVL3 is 55.7%; the similarity of IBR between DVL2 and DVL3 is 53.7%; the similarity of DBR between IQGAP1 and IQGAP2 is 82.4%; the similarity of DBR between IQGAP1 and IQGAP3 is 76.8%; the similarity of DBR between IQGAP2 and IQGAP3 is 72.8%. We conclude that IQGAP1 can associate with DVL in mammalian cells.

### IQGAP1 determines the nuclear localization of DVL

**Figure 2:**
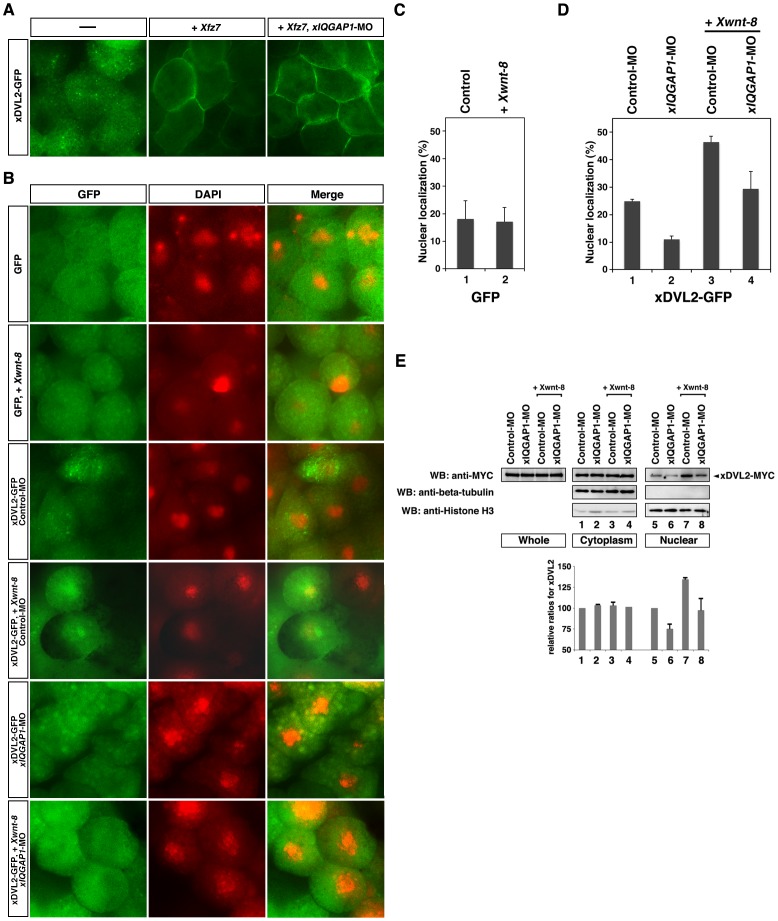
Localization of xDVL2-GFP. (**a,b**) Localization of xDVL2-GFP in *Xenopus* animal cap cells at stage 10. (**A**) xDvl2-GFP localized in punctate structures in the cytoplasm (left). Over-expression of *Xfz7* recruited xDVL2-GFP to the plasma membrane (center). *xIQGAP1*-MO did not affect the membrane localization of xDVL2-GFP induced by *Xfz7* (right). (**B**) Nuclear localization of GFP and xDVL2-GFP caused by over-expression of *Xwnt-8* and *xIQGAP1*-MO. GFP signals (left). DAPI staining of animal cap cells (center). Merge (right). (**C, D**) The ratio of cells that had nuclear fluorescence signals. The average of ratio was taken with six explants in 3 independent experiments (See Materials and Methods). Error bars represent standard deviation of the mean with six explants. Statistical significance was determined by Student's *t*-test. (**C**) The ratio of GFP localized in the nucleus in cells. *Xwnt-8* was co-injected in lanes 2. Lane 1: n = 291, 18.2%, lane 2: n = 320, 17.2%. P>0.1 [between lane 1 and lane 2]. (**D**) The ratio of xDVL2-GFP localized in the nucleus in cells injected with *xIQGAP1*-MO. *Xwnt-8* was co-injected in lanes 3 and 4. Lane 1: n = 918, 26.0%, lane 2: n = 1694, 11.0%, lane 3: n = 263, 46.4%, lane 4: n = 477, 29.4%. P<0.01 [between lane 1 and lane 2], P<0.01 [between lane 3 and lane 4]. (**E**) Cytoplasmic and nuclear distribution of xDVL2, xIQGAP1 and ß-catenin in animal cap cells. MYC-tagged *xDVL2* mRNA (100 pg) was injected into the animal poles of 4-cell stage embryos, and the injected animal caps were dissected at stage 10. Lysates from the animal caps were fractionated and subjected to Western blotting with indicated antibodies. Each relative intensity was measured by ImageJ, and its relative ratio was calculated against Input with beta-tubulin for cytoplasm or with Histone H3 for nuclear. Error bars represent standard deviation of the mean in three experiments. Statistical significance was determined by Student's *t*-test. P<0.1 [between lane 5 and lane 6], P<0.1 [between lane 7 and lane 8].

Whereas membrane-localized DVL functions to inhibit degradation of cytoplasmic ß-catenin in the canonical Wnt pathway [20], [21], nuclear-localized DVL is required, together with nuclear ß-catenin, for transactivation of the downstream targets of Wnt signaling [24]. To analyze DVL and IQGAP1 functions, we used the systems of *Xenopus* embryos. All isoforms of DVL and IQGAP are conserved well among vertebrates. The transcripts of their *Xenopus* homologues were expressed during early embryonic stages and were equivalently expressed at early gastrula stages (Fig. S1A and S1B). We subcloned their cDNA and generated their antisense morpholino oligo nucleotides (Fig. S1C). To examine how IQGAP affects DVL localization in Wnt signaling, we investigated the subcellular distribution of DVL fused to green fluorescent protein (GFP) in *Xenopus* embryonic cells. Fluorescence produced by the DVL2 (xDVL2)-GFP fusion appeared as a punctate pattern in the cytoplasm (Fig. 2A, left panel). DVL has been reported to be recruited to the plasma membrane by the Frizzled receptors in the Wnt pathway [21]. We confirmed that xDVL2-GFP accumulated in the plasma membrane when co-expressed with *Xenopus frizzled 7* (*Xfz7)* (Fig. 2A, center panel). Depletion of xIQGAP1 by antisense morpholino oligonucleotides (*xIQGAP1*-MO) did not affect the membrane localization of xDVL2-GFP (Fig. 2A, right panel). These results suggest that xIQGAP1 is not involved in the plasma membrane localization of xDVL2.

**Figure 3:**
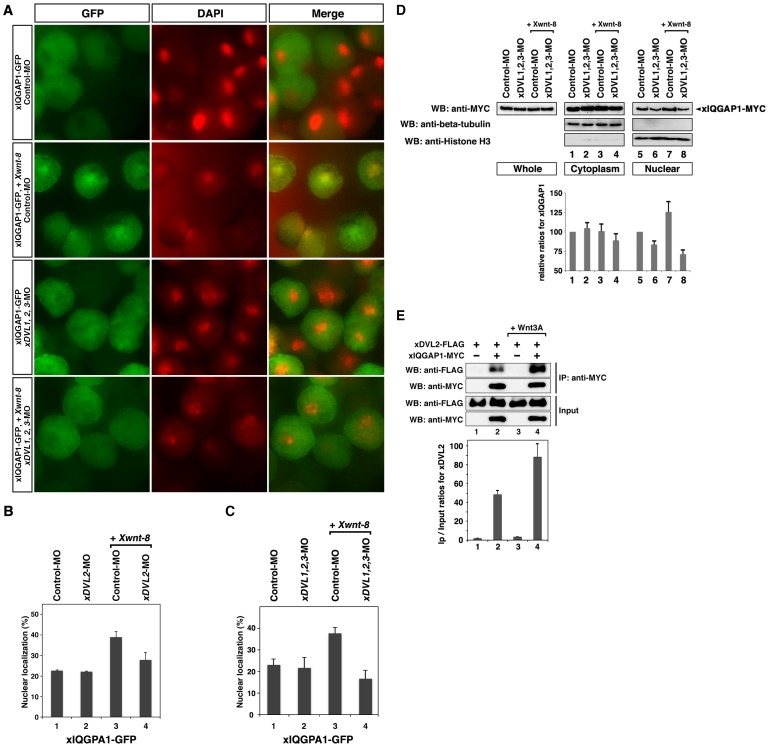
Localization of xIQGAP1-GFP. (**A**) Nuclear localization of xIQGAP1-GFP in stage 10 *Xenopus* animal cap cells over-expressing *Xwnt-8* and *xDVL1, 2, 3*-MO. GFP signals (left). DAPI staining of animal cap cells (center). Merge (right). (**B, C**)The ratio of cells that had nuclear fluorescence signals. The following procedure is indicated in Figure 2C. (**B**) The ratio of nuclear localized xIQGAP1-GFP in cells injected with *xDVL2*-MO. Lane 1: n = 388, 22.7%, lane 2: n = 361, 22.2%, lane 3: n = 616, 20.9%, lane 4: n = 534, 39.7%. P>0.1 [between lane 1 and lane 2], P<0.05 [between lane 3 and lane 4]. (**C**) The ratio of nuclear-localized xIQGAP1-GFP in cells injected with *xDVL1*-MO, *xDVL2*-MO and *xDVL3*-MO in *Xenopus* animal cap cells at stage 10. Lane 1: n = 1424, 23.0%, lane 2: n = 1306, 21.6%, lane 3: n = 1702, 37.6%, lane 4: n = 1409, 16.6%. P>0.1 [between lane 1 and lane 2], P<0.01 [between lane 3 and lane 4]. (**D**) Cytoplasmic and nuclear distribution of xIQGAP1 in animal cap cells. MYC-tagged *xIQGAP1* mRNA (100 pg) was injected into the animal poles of 4-cell stage embryos. The following procedure is indicated in Figure 2C. P<0.1 [between lane 5 and lane 6], P<0.1 [between lane 7 and lane 8]. (**E**) Interaction between ectopically-expressed xDVL2 and xIQGAP1 in HEK 293T cells. The transfected cultured cells were stimulated with recombinant human Wnt3A for 6 hours in lanes 3 and 4. The bars represent the IP/Input ratios of xDVL2-FLAG for each transfection. Error bars represent standard deviation of the mean in three experiments. Statistical significance was determined by Student's *t*-test. P<0.1 [between lane 2 and lane 4].

Stimulation by Wnt ligands is known to increase the nuclear localization of DVL [40]. When *xDVL2-GFP* was co-expressed with *Xwnt-8* in animal cap cells, nuclear GFP fluorescence was increased (Fig. 2B, third and fourth panels, 2D, lanes 1, 3), whereas GFP was mainly localized in the cytoplasm with or without *Xwnt-8* (Fig. 2B, first and second panels, 2C). However, injection of *xIQGAP1*-MO decreased nuclear fluorescence generated by co-expression of *xDVL2-GFP* and *Xwnt-8* (Fig. 2B, fifth and sixth panels, 2D, lanes 3, 4). We also confirmed that the amounts of xDVL2-MYC protein in the nuclear fractions of animal cap cells were reduced by depletion of xIQGAP1 (Fig. 2E). Expression of xIQGAP1-GFP resulted in fluorescence localized mainly to the cytoplasm, but nuclear fluorescence was increased by co-expression of *Xwnt-8* (Fig. 3A, first and second panels, 3B, lanes 1,3). Depletion of xDVL2 led to a decrease in nuclear fluorescence generated by the co-expression of *xIQGAP1-GFP* and *Xwnt-8* (Fig. 3B, lanes 3, 4). Moreover, we found that depletion of all three xDVLs (xDVL1, xDVL2 and xDVL3) reduced severely the nuclear localization of xIQGAP1-GFP in Wnt-stimulated cells (Fig. 3A, third and fourth panels, 3C, lanes 3, 4 and 3D). These results suggest that xIQGAP1 and xDVL2 play a crucial role in each other nuclear accumulation, depending on Wnt signaling.

We next examined whether a physical interaction between xIQGAP1 and xDVL2 is required for their nuclear localization. We generated a fusion of GFP to xDVL2-*Δ*IBR, a truncated version of xDVL2 lacking the IBR domain, and observed a punctate fluorescence pattern in the cytoplasm, similar to that seen with xDVL2-GFP (Fig. S2A). The proportion of fluorescence found in the nucleus was also similar to xDVL2-GFP (Fig. S2B). However, co-expression of *Xwnt-8* did not alter the proportion of GFP fluorescence found in the nucleus, (Fig. S2B), suggesting that the ability of xIQGAP1 to promote nuclear localization of xDVL2 requires the IBR domain in xDVL2. Consistent with this, a fusion of just the IBR domain to GFP (xDVL2-IBR-GFP) was localized predominantly in the nucleus (Fig. S2A and S2C). We next fused GFP to xIQGAP1-*Δ*DBR, which deletes the DBR domain of xIQGAP1. This xIQGAP1-*Δ*DBR-GFP fusion was also localized mainly in the cytoplasm (Fig. S2A), and the proportion of fluorescence found in the nucleus was less than that observed with full-length xIQGAP1-GFP (Fig. S2D). Co-expression of *Xwnt-8* also did not affect the nuclear localization of xIQGAP1-*Δ*DBR-GFP (Fig. S2D). A fusion of just the DBR domain to GFP (xIQGAP1-DBR-GFP) was localized mainly in the nucleus (Fig. S2A and S2E). We further investigated the effects of over-expression of xIQGAP1 or xDVL2 on the nuclear localization of xDVL2-GFP or xIQGAP1-GFP, respectively. Nuclear localization of xDVL2-GFP was increased by the expression of *xIQGAP1* and *xIQGAP1-DBR* (Fig. S3A). Meanwhile, expression of *xIQGAP1-* *Δ* *DBR* suppressed the nuclear localization of xDVL2-GFP induced by co-expression of *Xwnt-8* (Fig. S3A). In contrast, the nuclear localization of xIQGAP1-GFP was not affected by the expression of xDVL2 or xDVL2-IBR (Fig. S3B). However, *xDVL2-* *Δ* *IBR* suppressed the nuclear localization of xDVL2-GFP induced by co-expression of *Xwnt-8* (Fig. S3B). Moreover, immunoprecipitation experiments showed that Wnt stimulation increased the interaction between xDVL2 and xIQGAP1 in HEK 293T cells (Fig. 3E). Taken together, these results suggest that a physical interaction between xDVL2 and xIQGAP1 is required for their nuclear localization induced by the canonical Wnt signaling pathway.

### xIQGAP1 is necessary for the canonical Wnt pathway

**Figure 4:**
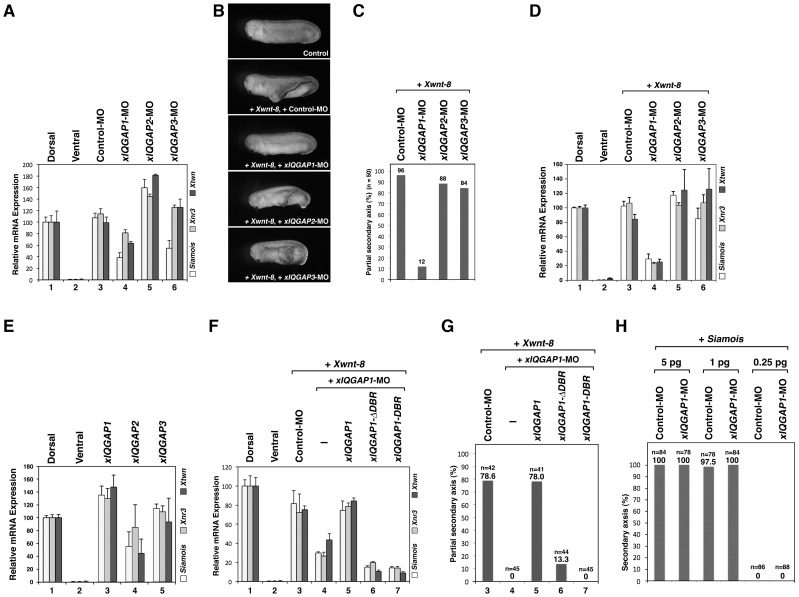
xIQGAP1 is required for the canonical Wnt pathway during early embryogenesis. (**A**) Quantitative RT-PCR analysis of early dorsal Wnt target genes (n = 3). Each indicated morpholino (15 ng) was injected into two dorsal blastomeres of 4-cell embryos. RNAs from dissected dorsal sectors of injected embryos were extracted at stage 10. RNAs from dissected dorsal and ventral sectors of uninjected embryos were used as controls. The value obtained for each gene was normalized to the level of ODC (ornithine decarboxylase). The value of dorsal sectors was set to 100 and other values were computed. Error bars represent standard deviation of the mean in three experiments. Statistical significance was determined by Student's *t*-test for each marker gene. The highest P values in three marker genes were chosen as a representative, as follows: P<0.05 [between lane 3 and lane 4], P<0.05 [between lane 3 and lane 5], P>0.1 [between lane 3 and lane 6]. (**B**) Phenotypes of injected embryos at stage 30. Control (upper panel). *Xwnt-8* mRNA (0.5 pg) was co-injected with *xIQGAP1*-, *xIQGAP2*- or *xIQGAP3*-MO (15 ng) into two ventral blastomeres of 4-cell embryos. (**C**) The ratio of injected embryos exhibiting a partial secondary axis. (**D**) Quantitative RT-PCR analysis of early dorsal Wnt target genes (n = 3). *xIQGAP1*-, *xIQGAP2*- or *xIQGAP3*-MO (15 ng) and *Xwnt-8* (0.5 pg) (20 pg) mRNA were ventrally co-injected. RNAs from dissected ventral sectors of injected embryos were extracted at stage 10. The following procedure is indicated in Figure 4A. P<0.01 [between lane 3 and lane 4], P<0.1 [between lane 3 and lane 5], P>0.1 [between lane 3 and lane 6]. (**E**) Quantitative RT-PCR analysis of early dorsal Wnt target genes (n = 3). *xIQGAP1*, *xIQGAP2* or *xIQGAP3* (400 pg) mRNA was dorsally injected. The following procedure is indicated in Figure 4A. P<0.05 [between lane 1 and lane 3], P<0.01 [between lane 1 and lane 4], P>0.1 [between lane 1 and lane 5]. (**F**) Quantitative RT-PCR analysis of early dorsal Wnt target genes (n = 3). *xIQGAP1*-MO (15 ng) and *Xwnt-8* (0.5 pg) (20 pg) mRNA were ventrally co-injected with *xIQGAP1* constructs: *xIQGAP1* (400 pg), *xIQGAP1-ΔDBR* (400 pg), *xIQGAP1-DBR* (400 pg) mRNA. RNAs from dissected ventral sectors of injected embryos were extracted at stage 10. The following procedure is indicated in Figure 4A. P<0.05 [between lane 3 and lane 4], P<0.05 [between lane 4 and lane 5], P<0.05 [between lane 5 and lane 6], P<0.05 [between lane 5 and lane 7]. (**G**) The ratio of injected embryos exhibiting a partial secondary axis. The numbered lanes indicate the injected mRNAs and MOs consistent with the numbering in Figure F. (**H**) The ratio of injected embryos that exhibited a secondary axis. Control-MO (15 ng) or *xIQGAP1*-MO (15 ng) was co-injected with *Siamois* mRNA (indicated dose).

To determine whether xIQGAP1 is also involved in the canonical Wnt pathway during early development, we investigated the effects of *xIQGAP1* on the transactivation of Wnt target genes and the secondary axis induction. Dorsal injection of an antisense morpholino oligonucleotide against xIQGAP1 (*xIQGAP1*-MO) reduced endogenous transcripts of the Wnt signal target genes *Siamois*, *Xnr3* and *Xtwn* (Fig. 4A). When *xDVL2*, *Xwnt-8* or *ß-catenin* mRNA was injected into the ventral sides of four-cell embryos, a secondary axis was formed and Wnt signal target genes were induced. The induction of the partial secondary axis and Wnt target genes induced by *Xwnt-8* was also suppressed by the depletion of xIQGAP1 (Fig. 4B–4D). Dorsal overexpression of *xIQGAP1* mRNA increased expression of Wnt target genes (Fig. 4E). On the other hand, the overexpression or depletion of *xIQGAP2* showed opposite effects for Wnt target gene expression (Fig. 4A, 4D and 4E). *xIQGAP3* showed ambiguous effects on Wnt target gene expression, especially expression of *Siamois* (Fig. 4A, 4D and 4E). However, depletions of either xIQGAP2 or xIQGAP3 did not alter the partial secondary axis induction by *Xwnt-8* (Fig. 4B and 4C). These results suggest that xIQGAP1 is necessary for Wnt-related early embryogenesis in a subtype-specific manner.

We also found that the suppressions of induction of Wnt target genes and partial secondary axis by *xIQGAP1*-MO were rescued by expression of wild-type *xIQGAP1*, but not by either *xIQGAP1-ΔDBR* or *-DBR* (Fig. 4F, 4G, S4A–S4D). However, depletion of xIQGAP1 did not affect the secondary axis formation induced by *Simaois*, which is one of the Wnt signal target genes (Fig. 4H). Moreover, we also observed the reduction of endogenous IQGAP1 by the siRNA (siIQGAP1) suppressed the expression of Wnt target genes induced by Wnt3A stimulation in cultured cells (Fig. S5A). These results suggest that xIQGAP1 functions as an intermediate molecule in the canonical Wnt signaling pathway in early development promoting the nuclear localization of xDVL2.

### The IQGAP binding region of xDVL2 is important for canonical Wnt signaling

**Figure 5:**
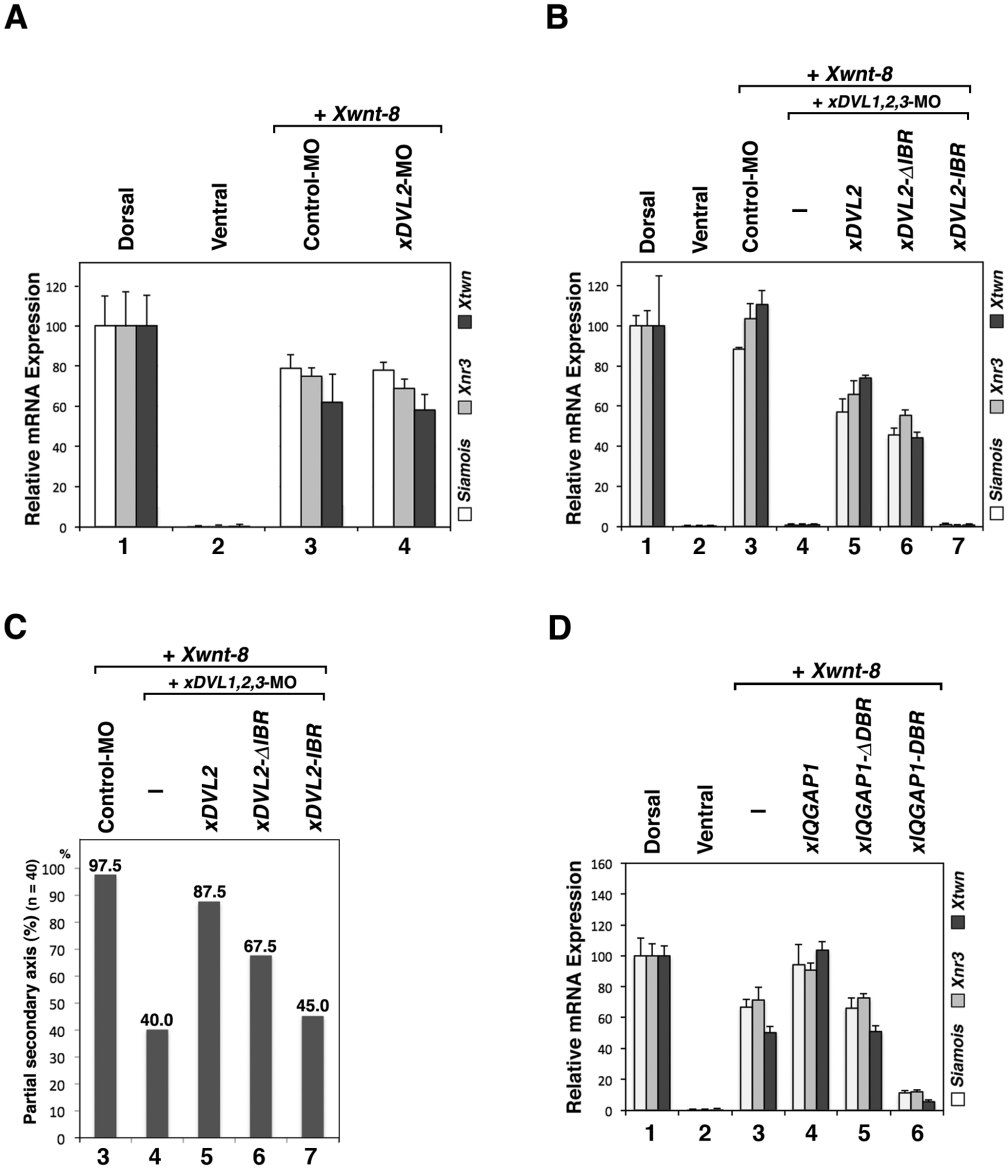
The role of the xIQGAP1-binding region of xDVL2 in canonical Wnt signaling during early embryogenesis. (**A**) Quantitative RT-PCR analysis of early dorsal Wnt target genes (n = 3). Control-MO (15 ng) or *xDVL2*-MO (15 ng) was ventrally co-injected with *Xwnt-8* (0.5 pg) mRNA. RNAs from dissected ventral sectors of injected embryos were extracted at stage 10. The following procedure is indicated in Figure 4A. Error bars represent standard deviation of the mean in three experiments. Statistical significance was determined by Student's *t*-test for each marker gene. The highest P values in three marker genes were chosen as a representative, as follows: P>0.1 [between lane 3 and lane 4]. (**B**) Quantitative RT-PCR analysis of early dorsal Wnt target genes (n = 3). *xDVL1*-MO (10 ng), *xDVL2*-MO (10 ng), *xDVL3*-MO (10 ng) and *Xwnt-8* (0.5 pg) mRNA were ventrally co-injected with *xDVL2* constructs: *xDVL2* (25 pg), *xDVL2-ΔIBR* (25 pg), *xDVL2-IBR* (25 pg) mRNA. RNAs from dissected ventral sectors of injected embryos were extracted at stage 10. The following procedure is indicated in Figure 4A. P<0.05 [between lane 3 and lane 4], P<0.05 [between lane 4 and lane 5], P<0.1 [between lane 5 and lane 6], P<0.05 [between lane 5 and lane 7]. (**C**) The ratio of injected embryos that exhibited a partial secondary axis. The numbered lanes indicate the injected mRNAs and MOs consistent with the numbering in Figure B. (**D**) Quantitative RT-PCR analysis of early dorsal Wnt target genes (n = 3). *Xwnt-8* (0.5 pg) mRNA was ventrally co-injected with *xIQGAP1* constructs: *xIQGAP1* (400 pg), *xIQGAP1-ΔDBR* (1 ng), *xIQGAP1-DBR* (1 ng) mRNA. The following procedure is indicated in Figure 4A. P<0.01 [between lane 3 and lane 4], P>0.1 [between lane 3 and lane 5], P<0.05 [between lane 3 and lane 6].

To further confirm whether the binding between IQGAP1 and DVL is critical in the canonical Wnt pathway during early development, we investigated the effects of *xDVL* mutants on the transactivation of Wnt target genes and secondary axis induction. Similar to our previous observation that injection of *xDVL2*-MO did not affect severely nuclear localization of xIQGAP1 (Fig. 3B), we also observed no reduction in Wnt target gene expression induced at the ventral side by *Xwnt-8* when *xDVL2*-MO was co-injected (Fig. 5A). However, depletion of all three xDVLs reduced nuclear localization of xIQGAP1, expression of the Wnt target genes and suppressed formation of the secondary axis induced by *Xwnt-8* or *ß-catenin* (Fig. 3C, 3D, 5B, and 5C). These results suggest that three xDVL genes act redundantly in the canonical Wnt signal pathway. Suppression of secondary axis formation and Wnt target gene expression caused by depletion of all three xDVLs could be rescued by co-expression of wild-type *xDVL2*, but only weakly by *xDVL2-ΔIBR* and barely by *xDVL2-IBR* (Fig. 5B, and 5C). Moreover, co-expression of *xIQGAP1-DBR* reduced the expression of Wnt target genes induced by *Xwnt-8*, *xDVL2* or *ß-catenin* in *Xenopus* embryos (Fig. 5D, S6A and S6B), and the expression of xIQGAP1-DBR in cultured cells reduced the expression of Wnt target gene induced by Wnt3A (Fig. S5B). These results support the idea that binding between xDVL2 and xIQGAP1 plays important roles for canonical Wnt signaling.

## Discussion

In the present studies, we show that IQGAP1 is necessary for the nuclear localization of DVL in the canonical Wnt signaling pathway. Previous studies have shown that nuclear localization of DVL is necessary for the sequential activation of Wnt target genes [24], [40]. It has also been shown that the nuclear localization signal (NLS) located between the PDZ and DEP domain of xDVL2, and the nuclear export signal (NES) located at the C-terminus are important for the nuclear localization and transcriptional activation of Wnt target genes [40]. Interestingly, we observed that xDVL2-IBR, a truncated protein consisting of just the IQGAP-binding region from the C-terminus of xDVL2, localized predominantly in the nucleus, even though this region contains an NES and not an NLS. Moreover, the nuclear localization of xDVL2-*Δ*IBR-GFP, which contains an NLS, but not an NES, did not increase with Wnt-8 stimulation any longer. Although our findings suggest a new molecular mechanism mediating xIQGAP1-dependent nuclear localization of xDVL2, we could not positively state that our findings is independent of the NLS or NES motifs within DVL2. Further studies need to clarify the inconsistencies using same mutants.

In the canonical Wnt pathway, DVL is necessary for both the inactivation of ß-catenin degradation in cytoplasm^21^ and the activation of Wnt target genes by forming a complex containing ß-catenin and Tcf in nuclei [24]. In the present study, we have shown that IQGAP1 interacted with DVL and that the depletion of IQGAP1 reduced the nuclear localization of DVL, while IQGAP1 did not affect on the membrane localization of DVL required for ß-catenin stability in cytoplasm. These results suggest that IQGAP1 plays a role in the nuclear translocation of DVL in the canonical Wnt pathway.

We showed that the nuclear localization of xIQGAP1 and xDVL2 were increased by Wnt stimulation. In contrast, the nuclear localization of xDVL2-*Δ*IBR-GFP and xIQGAP1-*Δ*DBR-GFP did not increase with Wnt stimulation, while over-expression of *xDVL2-ΔIBR* or *xIQGAP1-ΔDBR* interfered with the nuclear localization of xIQGAP1-GFP or xDVL2-GFP induced by Wnt stimulation, respectively. Reduced expression of Wnt target genes due to depletion of endogenous xIQGAP1 or xDVLs was barely or weakly rescued by expression of *xIQGAP1-ΔDBR* or *xDVL2-ΔIBR*, respectively. Conversely, xDVL2-IBR-GFP and xIQGAP1-DBR-GFP were mainly localized in the nuclei regardless of Wnt stimulation. Moreover, *xIQGAP1-DBR* reduced expression of the Wnt target genes induced by *xDVL2*, *Xwnt-8* and *ß-catenin*. Taken together, these results suggest that the domains mediating binding between xIQGAP1 and xDVL2 play important roles in both their nuclear localization and their Wnt-stimulated activities.

In vertebrates, DVL1, DVL2 and DVL3 have redundant function in part [51], [52]. Depletion of all three xDVLs; xDVL1, xDVL2 and xDVL3, did reduce severely the nuclear localization of xIQGAP1 rather than only xDVL2 depletion. Moreover, induction of Wnt target genes and formation of the secondary axis by *Xwnt-8* or *ß-catenin* was suppressed by the depletion of all three xDVLs, but not by the depletion of xDVL2 alone. However, xDVL2 expression could rescue suppression of Wnt target genes by the depletion of all three xDVLs. These results suggest that xDVL1, xDVL2 and xDVL3 also function redundantly in Wnt signaling involving xIQGAP1. On the other hand, we showed that all IQGAP isoforms bound to each DVL isoform, nevertheless only IQGAP1 was necessary for Wnt signaling. Previous report also showed the functional differences, their subcellular localization and the interaction with binding proteins among IQGAP isoforms in many different cellular processes [36]. Therefore, unidentified binding molecules might cause the functional differences among IQGAP isoforms. Further molecular analyses will be needed to clarify the different roles of IQGAP isoforms.

## Supporting Information

Figure S1 **Expression of** ***Xenopus*** **DVL and IQGAP1 isoforms and confirmation of the morpholino specificity.** Reverse transcription–polymerase chain reaction analysis was performed using total RNA extracted from *Xenopus* embryos at different stages of development and from different regions. *Ornithine decarboxylase* (*ODC*) was used as an internal control. (**A**) Temporal expression patterns. U, unfertilized eggs. The numbers indicate developmental stages. (**B**) Spatial expression patterns. Embryos were dissected at stage 10, and dissections were performed as shown in the right panel. D, dorsal; Vn, ventral; A, animal; M, marginal; Vg, vegetal; H, head. (**C**) Morpholino (MO) (10 ng) and FLAG-tagged mRNAs (100 pg) were co-injected with *ß-globin-FLAG* mRNA (100 pg) as loading control into the animal poles of 4-cell stage embryos, and the injected animal caps were dissected at stage 10. Lysates from the animal caps were subjected to Western blotting with anti-FLAG antibody (M2, Sigma).
(TIF)

Figure S2 **Localization of xDVL2 and xIQGAP1 GFP constructs in** ***Xenopus*** **animal cap cells at stage 10.** (**A**) GFP signals (left panels). DAPI staining (center panels). Merge (right panels). xDVL2-*Δ*IBR-GFP (upper panels). xDVL2-IBR-GFP (second panels). xIQGAP1-*Δ*DBR-GFP (third panels). xIQGAP1-DBR-GFP (bottom panels). (**B-E**) The ratio of cells that had nuclear fluorescence signals. The average of ratio was taken with six explants in 3 independent experiments (See Materials and Methods). Error bars represent standard deviation of the mean with six explants. Statistical significance was determined by Student's *t*-test. (**B**) The ratio of nuclear-localized xDVL2-*Δ*IBR-GFP. Lane 1: n = 499, 22.4%, lane 2: n = 349, 23.8%. P>0.1 [between lane 1 and lane 2]. (**C**) The ratio of nuclear-localized xDVL2-IBR-GFP. Lane 1: n = 740, 78.9%, lane 2: n = 420, 87.6%. P>0.1 [between lane 1 and lane 2]. (**D**) The ratio of nuclear localized xIQGAP1-*Δ*DBR-GFP. Lane 1: n = 1205, 13.0%, lane 2: n = 410, 14.6%. P>0.1 [between lane 1 and lane 2]. (**e**) The ratio of nuclear localized xIQGAP1-DBR-GFP. Lane 1: n = 1598, 80.6%, lane 2: n = 408, 92.7%. P<0.01 [between lane 1 and lane 2].
(TIF)

Figure S3 **Effects of over-expression of** ***xIQGAP1*** **and** ***xDVL2*** **constructs on the nuclear localization of xDVL2-GFP and xIQGAP1-GFP.** The ratio of cells that had nuclear fluorescence signals. The average of ratio was taken with six explants in 3 independent experiments (See Materials and Methods). Error bars represent standard deviation of the mean with six explants. Statistical significance was determined by Student's *t*-test. (**A**) The ratio of nuclear-localized xDVL2-GFP in cells expressing various *xIQGAP1* constructs: *xIQGAP1*, *xIQGAP1-ΔDBR* or *xIQGAP1-DBR* mRNA. Lane 1: n = 1038, 22.1%, lane 2: n = 495, 31.1%, lane 3: n = 698, 26.5%, lane 4: n = 262, 55.7%, lane 5: n = 1171, 41.8%, lane 6: n = 655, 52.7%, lane 7: n = 611, 27.7%, lane 8: n = 520, 61.0%. P<0.1 [between lane 1 and lane 2], P>0.1 [between lane 1 and lane 3], P<0.01 [between lane 1 and lane 4], P<0.01 [between lane 5 and lane 6], P<0.01 [between lane 5 and lane 7], P<0.01 [between lane 5 and lane 8]. (**B**) The ratio of nuclear localized xIQGAP1-GFP in cells expressing various *xDVL2* constructs: *xDVL21*, *xDVL21-ΔIBR* or *xDVL2-IBR* mRNA. Lane 1: n = 801, 22.0%, lane 2: n = 726, 23.3%, lane 3: n = 765, 21.4%, lane 4: n = 1223, 22.2%, lane 5: n = 1171, 36.5%, lane 6: n = 362, 37.6%, lane 7: n = 641, 22.3%, lane 8: n = 549, 33.2%. P>0.1 [between lane 1 and lane 2], P>0.1 [between lane 1 and lane 3], P>0.1 [between lane 1 and lane 4], P>0.1 [between lane 5 and lane 6], P<0.01 [between lane 5 and lane 7], P>0.1 [between lane 5 and lane 8].
(TIF)

Figure S4 **The effects of xIQGAP isoforms and xIQGAP1 mutated constructs.** (**A, C**) Quantitative RT-PCR analysis of early dorsal Wnt target genes (n = 3). *xIQGAP1*-MO (15 ng) and *xDVL2* (50 pg) or *ß-catenin* (20 pg) mRNA were ventrally co-injected with *xIQGAP1* constructs: *xIQGAP1* (400 pg), *xIQGAP1-ΔDBD* (400 pg), *xIQGAP1-DBD* (400 pg) mRNA. RNAs from dissected ventral sectors of injected embryos were extracted at stage 10. RNAs from dissected dorsal and ventral sectors of uninjected embryos were used as controls. The value obtained for each gene was normalized to the level of ODC (ornithine decarboxylase). The value of dorsal sectors was set to 100 and other values were computed. Error bars represent standard deviation of the mean in three experiments. Statistical significance was determined by Student's *t*-test for each marker gene. The highest P values in three marker genes were chosen as a representative. (**A**) P<0.05 [between lane 3 and lane 4], P<0.05 [between lane 4 and lane 5], P<0.05 [between lane 5 and lane 6], P<0.05 [between lane 5 and lane 7]. (**C**) P<0.05 [between lane 3 and lane 4], P<0.05 [between lane 4 and lane 5], P<0.05 [between lane 5 and lane 6], P<0.05 [between lane 5 and lane 7]. (**B, D**) The ratio of injected embryos exhibiting a partial secondary axis. The numbered lanes indicate the injected mRNAs and MOs consistent with the numbering in Figure **A** and **C**, respectively.
(TIF)

Figure S5 **The effects of** ***IQGAP1*** **on the Wnt target genes in cultured cells.** RT-PCR analysis of Wnt target genes in NIH3T3 cells. The transfected cultured cells were stimulated with the Wnt-3A conditioned medium from L-Wnt-3A cells for 24 hours. The condition medium from L cells was used for unstimulated control. GAPDH was used for normalization of cDNA samples. (**A**) siRNAs were transfected. (**B**) xIQGAP1 or xIQGAP1-DBD was transfected.
(TIF)

Figure S6 **The effects of xDVL2 and xIQGAP1 mutated constructs.** (**A, B**) Quantitative RT-PCR analysis of early dorsal Wnt target genes (n = 3). *xDVL2* (50 pg) or *ß-catenin* (20 pg) mRNA were ventrally co-injected with *xIQGAP1* constructs: *xIQGAP1* (400 pg), *xIQGAP1-ΔDBD* (1 ng), *xIQGAP1-DBD* (1 ng) mRNA. The following procedure is indicated in Figure S4A. (**A**) P<0.1 [between lane 3 and lane 4], P<0.05 [between lane 3 and lane 5], P<0.05 [between lane 3 and lane 6]. (**B**) P<0.05 [between lane 3 and lane 4], P>0.1 [between lane 3 and lane 5], P<0.05 [between lane 3 and lane 6].
(TIF)
