# Supplementary figures and images for: IQGAP1 Functions as a Modulator of Dishevelled Nuclear Localization in Wnt Signaling
Source: PLoS One. 2013 Apr 5;8(4):e60865. doi: 10.1371/journal.pone.0060865 (PMC3618174; doi:10.1371/journal.pone.0060865)

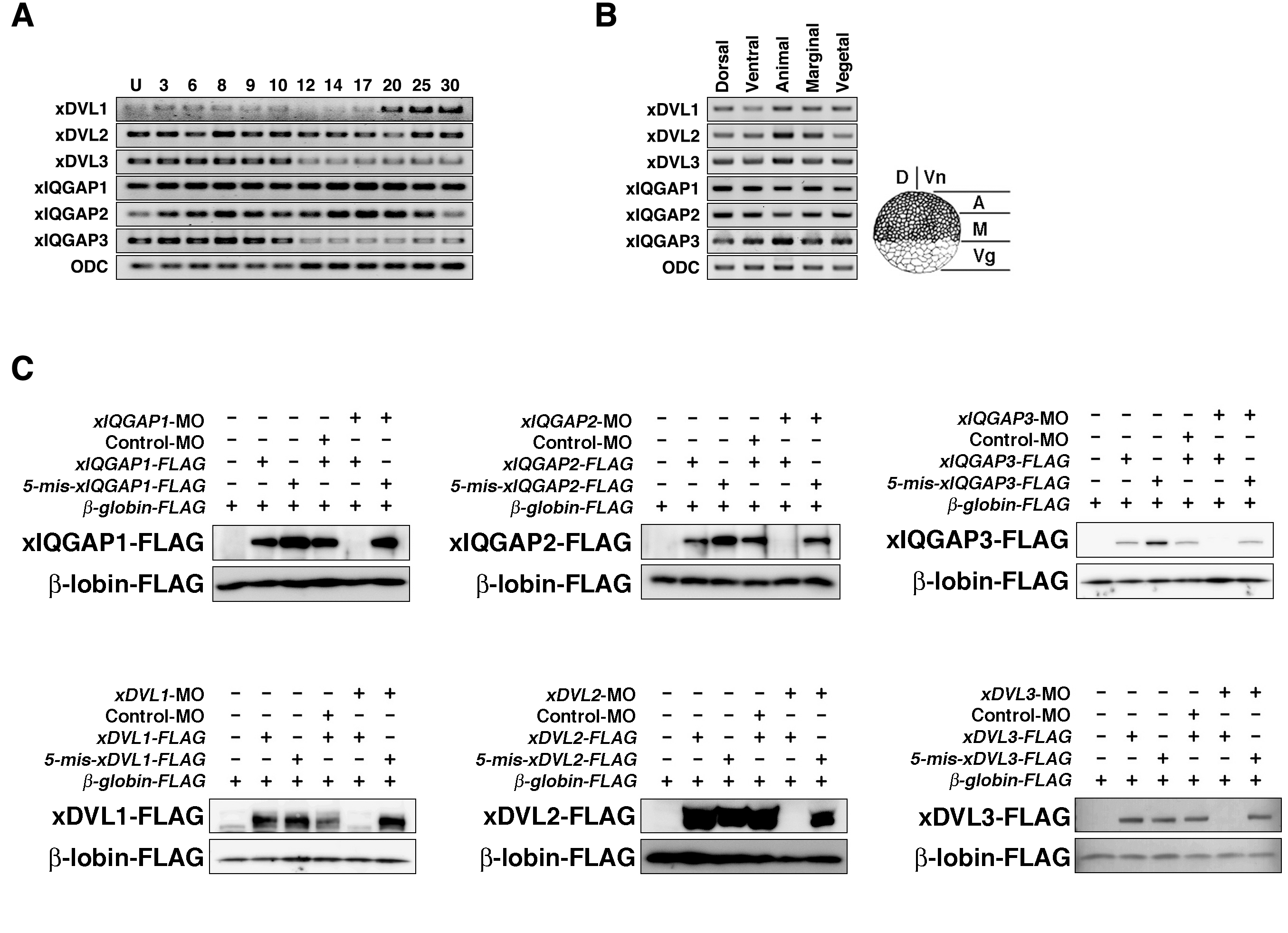

Supplement: Figure S1 — Expression of Xenopus DVL and IQGAP1 isoforms and confirmation of the morpholino specificity. Reverse transcription–polymerase chain reaction analysis was performed using total RNA extracted from Xenopus embryos at different stages of development and from different regions. Ornithine decarboxylase (ODC) was used as an internal control. (A) Temporal expression patterns. U, unfertilized eggs. The numbers indicate developmental stages. (B) Spatial expression patterns. Embryos were dissected at stage 10, and dissections were performed as shown in the right panel. D, dorsal; Vn, ventral; A, animal; M, marginal; Vg, vegetal; H, head. (C) Morpholino (MO) (10 ng) and FLAG-tagged mRNAs (100 pg) were co-injected with ß-globin-FLAG mRNA (100 pg) as loading control into the animal poles of 4-cell stage embryos, and the injected animal caps were dissected at stage 10. Lysates from the animal caps were subjected to Western blotting with anti-FLAG antibody (M2, Sigma). (TIF) [file pone.0060865.s001.tif]

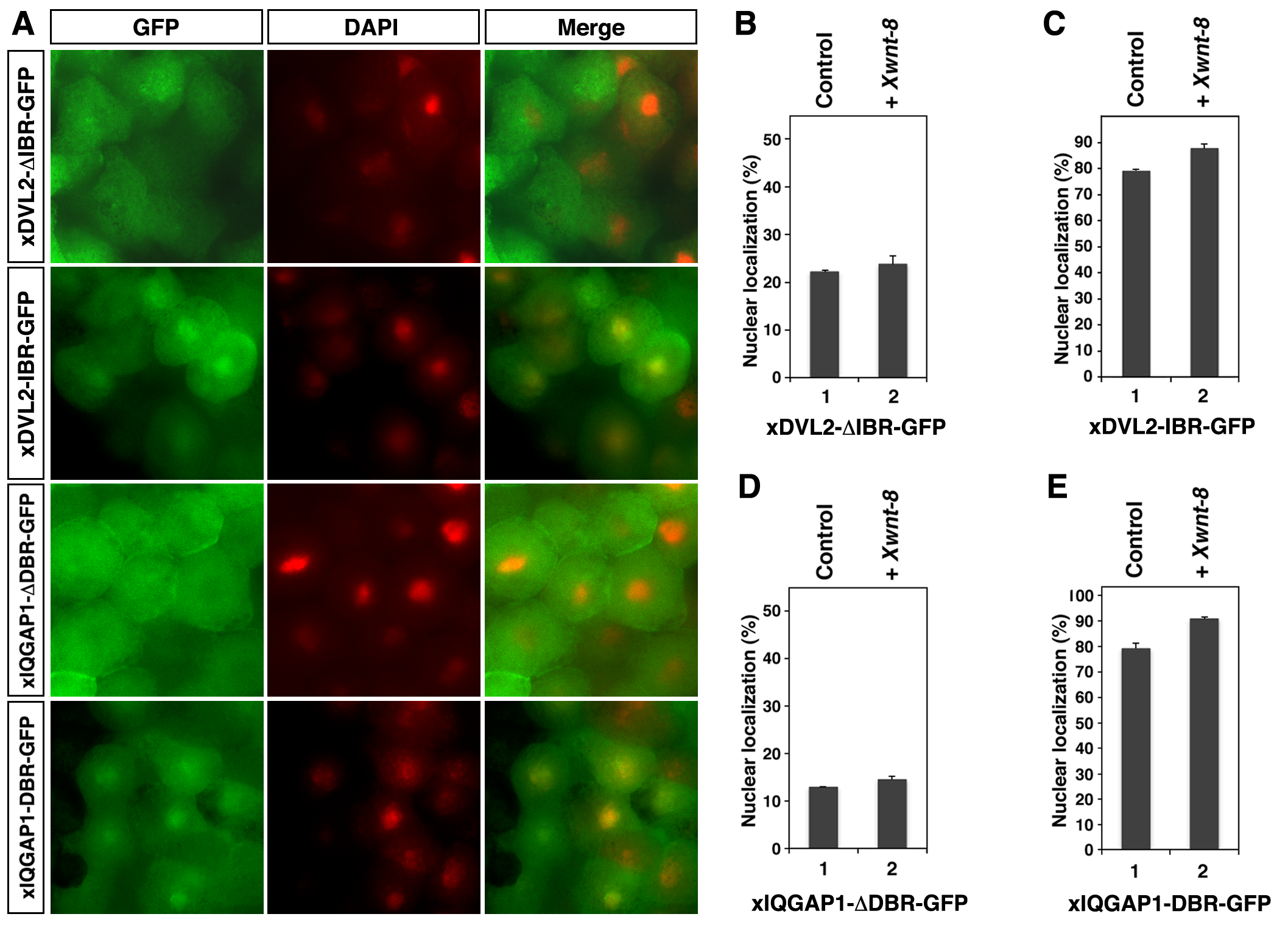

Supplement: Figure S2 — Localization of xDVL2 and xIQGAP1 GFP constructs in Xenopus animal cap cells at stage 10. (A) GFP signals (left panels). DAPI staining (center panels). Merge (right panels). xDVL2-ΔIBR-GFP (upper panels). xDVL2-IBR-GFP (second panels). xIQGAP1-ΔDBR-GFP (third panels). xIQGAP1-DBR-GFP (bottom panels). (B-E) The ratio of cells that had nuclear fluorescence signals. The average of ratio was taken with six explants in 3 independent experiments (See Materials and Methods). Error bars represent standard deviation of the mean with six explants. Statistical significance was determined by Student's t-test. (B) The ratio of nuclear-localized xDVL2-ΔIBR-GFP. Lane 1: n = 499, 22.4%, lane 2: n = 349, 23.8%. P>0.1 [between lane 1 and lane 2]. (C) The ratio of nuclear-localized xDVL2-IBR-GFP. Lane 1: n = 740, 78.9%, lane 2: n = 420, 87.6%. P>0.1 [between lane 1 and lane 2]. (D) The ratio of nuclear localized xIQGAP1-ΔDBR-GFP. Lane 1: n = 1205, 13.0%, lane 2: n = 410, 14.6%. P>0.1 [between lane 1 and lane 2]. (e) The ratio of nuclear localized xIQGAP1-DBR-GFP. Lane 1: n = 1598, 80.6%, lane 2: n = 408, 92.7%. P<0.01 [between lane 1 and lane 2]. (TIF) [file pone.0060865.s002.tif]

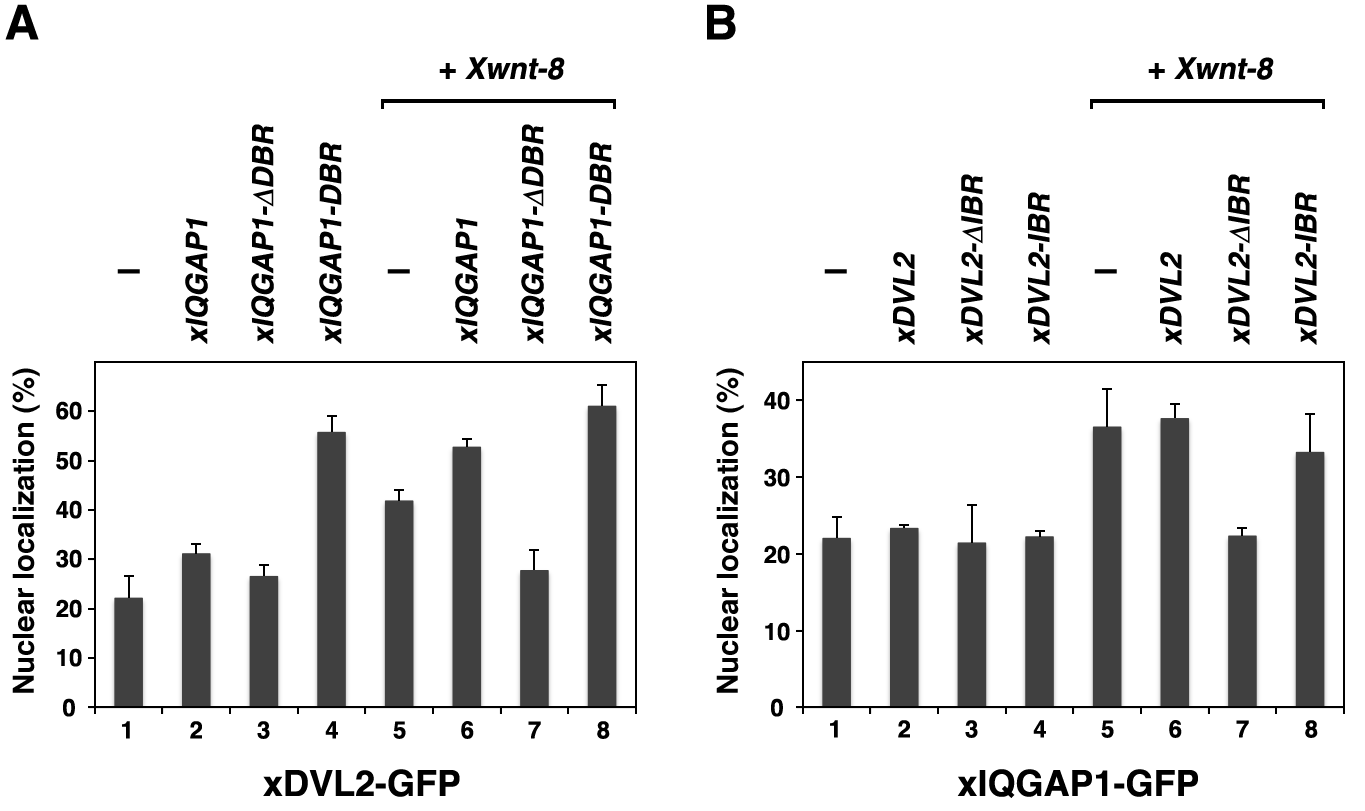

Supplement: Figure S3 — Effects of over-expression of xIQGAP1 and xDVL2 constructs on the nuclear localization of xDVL2-GFP and xIQGAP1-GFP. The ratio of cells that had nuclear fluorescence signals. The average of ratio was taken with six explants in 3 independent experiments (See Materials and Methods). Error bars represent standard deviation of the mean with six explants. Statistical significance was determined by Student's t-test. (A) The ratio of nuclear-localized xDVL2-GFP in cells expressing various xIQGAP1 constructs: xIQGAP1, xIQGAP1-ΔDBR or xIQGAP1-DBR mRNA. Lane 1: n = 1038, 22.1%, lane 2: n = 495, 31.1%, lane 3: n = 698, 26.5%, lane 4: n = 262, 55.7%, lane 5: n = 1171, 41.8%, lane 6: n = 655, 52.7%, lane 7: n = 611, 27.7%, lane 8: n = 520, 61.0%. P<0.1 [between lane 1 and lane 2], P>0.1 [between lane 1 and lane 3], P<0.01 [between lane 1 and lane 4], P<0.01 [between lane 5 and lane 6], P<0.01 [between lane 5 and lane 7], P<0.01 [between lane 5 and lane 8]. (B) The ratio of nuclear localized xIQGAP1-GFP in cells expressing various xDVL2 constructs: xDVL21, xDVL21-ΔIBR or xDVL2-IBR mRNA. Lane 1: n = 801, 22.0%, lane 2: n = 726, 23.3%, lane 3: n = 765, 21.4%, lane 4: n = 1223, 22.2%, lane 5: n = 1171, 36.5%, lane 6: n = 362, 37.6%, lane 7: n = 641, 22.3%, lane 8: n = 549, 33.2%. P>0.1 [between lane 1 and lane 2], P>0.1 [between lane 1 and lane 3], P>0.1 [between lane 1 and lane 4], P>0.1 [between lane 5 and lane 6], P<0.01 [between lane 5 and lane 7], P>0.1 [between lane 5 and lane 8]. (TIF) [file pone.0060865.s003.tif]

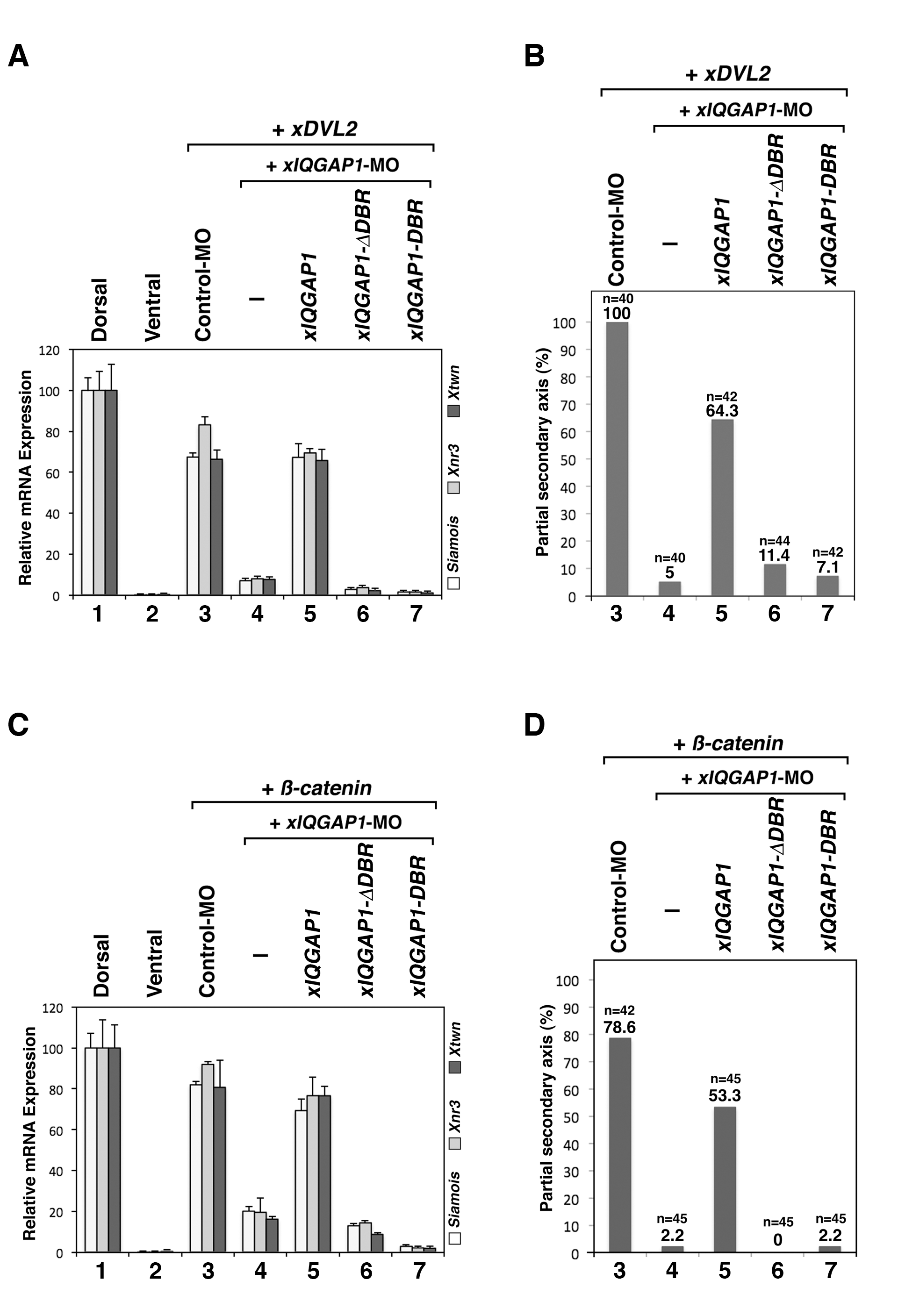

Supplement: Figure S4 — The effects of xIQGAP isoforms and xIQGAP1 mutated constructs. (A, C) Quantitative RT-PCR analysis of early dorsal Wnt target genes (n = 3). xIQGAP1-MO (15 ng) and xDVL2 (50 pg) or ß-catenin (20 pg) mRNA were ventrally co-injected with xIQGAP1 constructs: xIQGAP1 (400 pg), xIQGAP1-ΔDBD (400 pg), xIQGAP1-DBD (400 pg) mRNA. RNAs from dissected ventral sectors of injected embryos were extracted at stage 10. RNAs from dissected dorsal and ventral sectors of uninjected embryos were used as controls. The value obtained for each gene was normalized to the level of ODC (ornithine decarboxylase). The value of dorsal sectors was set to 100 and other values were computed. Error bars represent standard deviation of the mean in three experiments. Statistical significance was determined by Student's t-test for each marker gene. The highest P values in three marker genes were chosen as a representative. (A) P<0.05 [between lane 3 and lane 4], P<0.05 [between lane 4 and lane 5], P<0.05 [between lane 5 and lane 6], P<0.05 [between lane 5 and lane 7]. (C) P<0.05 [between lane 3 and lane 4], P<0.05 [between lane 4 and lane 5], P<0.05 [between lane 5 and lane 6], P<0.05 [between lane 5 and lane 7]. (B, D) The ratio of injected embryos exhibiting a partial secondary axis. The numbered lanes indicate the injected mRNAs and MOs consistent with the numbering in Figure A and C, respectively. (TIF) [file pone.0060865.s004.tif]

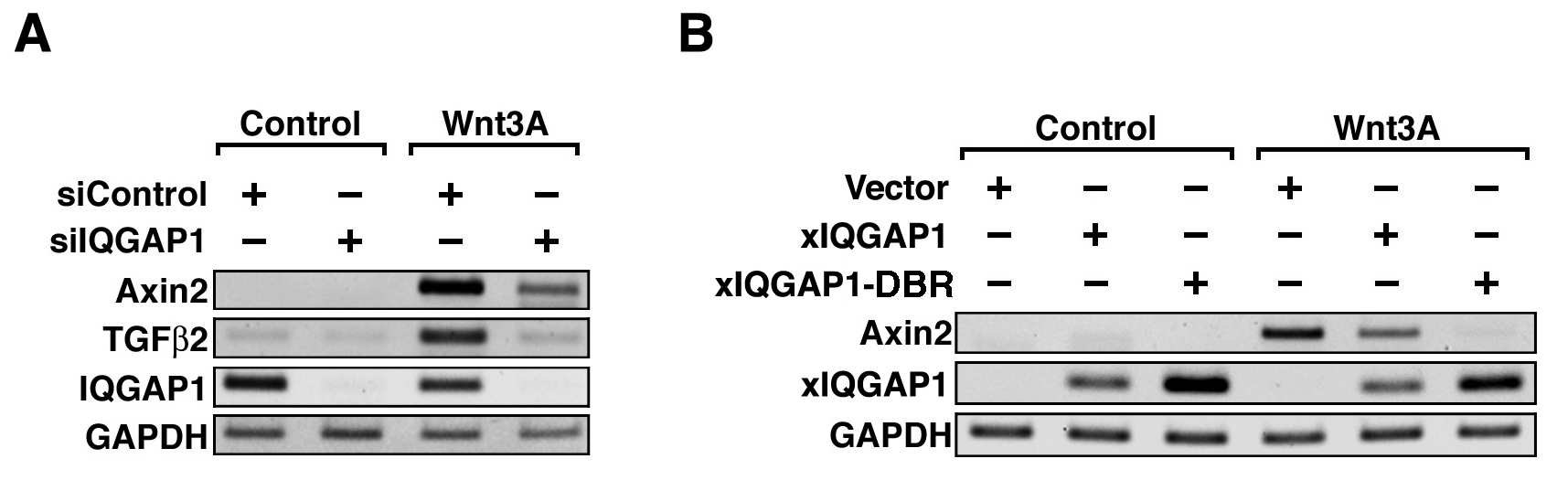

Supplement: Figure S5 — The effects of IQGAP1 on the Wnt target genes in cultured cells. RT-PCR analysis of Wnt target genes in NIH3T3 cells. The transfected cultured cells were stimulated with the Wnt-3A conditioned medium from L-Wnt-3A cells for 24 hours. The condition medium from L cells was used for unstimulated control. GAPDH was used for normalization of cDNA samples. (A) siRNAs were transfected. (B) xIQGAP1 or xIQGAP1-DBD was transfected. (TIF) [file pone.0060865.s005.tif]

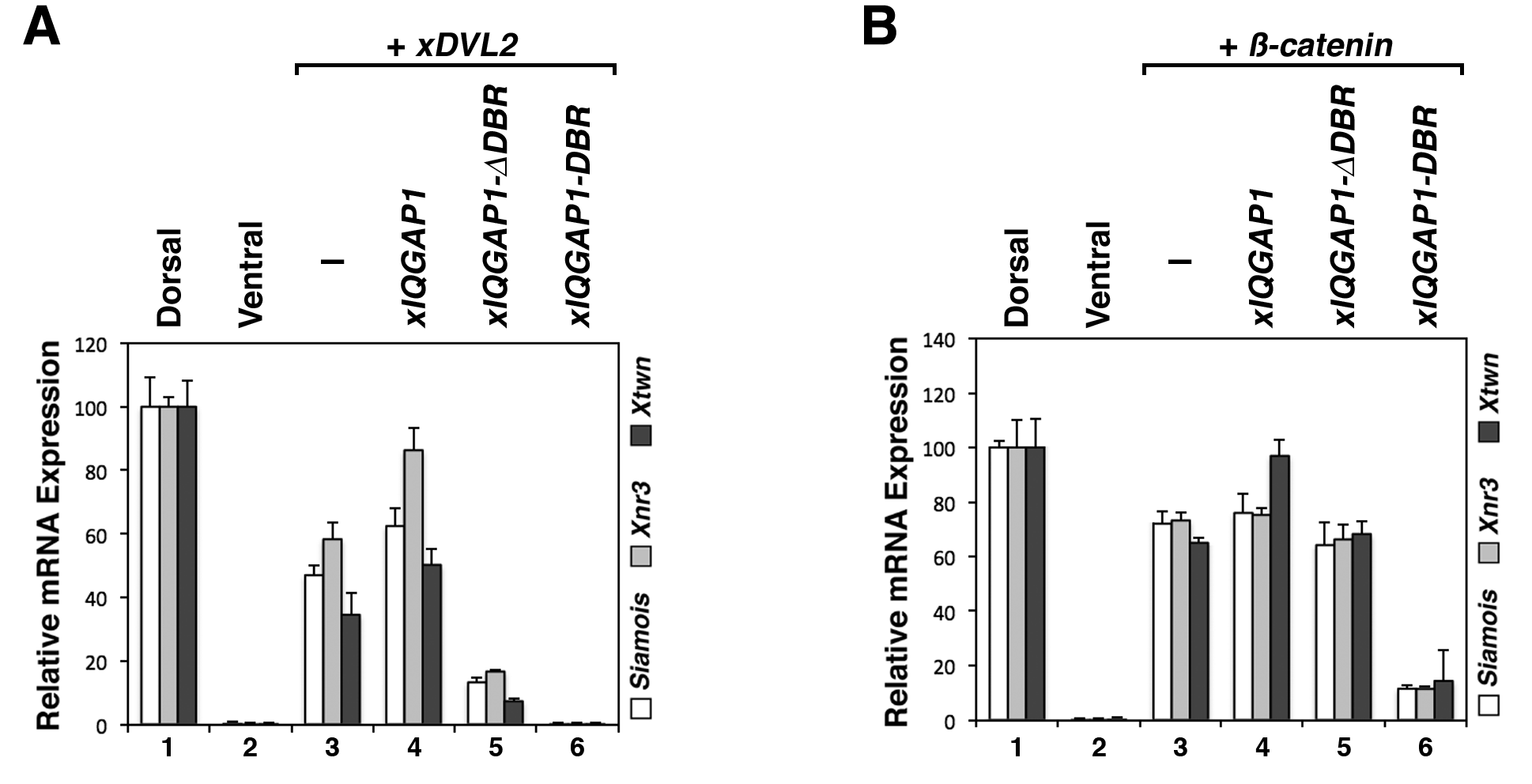

Supplement: Figure S6 — The effects of xDVL2 and xIQGAP1 mutated constructs. (A, B) Quantitative RT-PCR analysis of early dorsal Wnt target genes (n = 3). xDVL2 (50 pg) or ß-catenin (20 pg) mRNA were ventrally co-injected with xIQGAP1 constructs: xIQGAP1 (400 pg), xIQGAP1-ΔDBD (1 ng), xIQGAP1-DBD (1 ng) mRNA. The following procedure is indicated in Figure S4A. (A) P<0.1 [between lane 3 and lane 4], P<0.05 [between lane 3 and lane 5], P<0.05 [between lane 3 and lane 6]. (B) P<0.05 [between lane 3 and lane 4], P>0.1 [between lane 3 and lane 5], P<0.05 [between lane 3 and lane 6]. (TIF) [file pone.0060865.s006.tif]
